# Supplementary material for: Lack of children in public medical imaging data points to growing age bias in biomedical AI
Source: medRxiv. 2025 Jun 10:2025.06.06.25328913. Preprint. [Version 2] doi: 10.1101/2025.06.06.25328913 (PMC12259205; doi:10.1101/2025.06.06.25328913)
Supplement: Supplement 2 [file NIHPP2025.06.06.25328913v2-supplement-2.pdf]

medRxiv preprint doi: https://doi.org/10.1101/2025.06.06.25328913; this version posted June 10, 2025. The copyright holder for this preprint (which was not certified by peer review) is the author/funder, who has granted medRxiv a license to display the preprint in perpetuity.
It is made available under a CC-BY 4.0 International license .

Supplementary Methods

A Case Study of Adult-Trained Cardiomegaly Classifiers on Pediatric Patients

Dataset Standardization. To standardize the adult datasets, the following steps are taken:

i. Exclude pediatric patients aged 17 and under, if any.
ii. Exclude data points without findings annotated or with particularly invalid age annotation.
iii. Keep only PA view images.
iv. Sample one image per patient, for patients with data from multiple visits, in priority of: **a)** having valid age annotated, and **b)** having cardiomegaly.
v. Exclude patients with findings other than cardiomegaly and patients with uncertain cardiomegaly findings.

Each of the four adult-only datasets are then split independently into a training set, calibration set and held-out set of healthy adults. To create the held-out set, 10% of healthy adults are sampled from each age bin in [18, 25, 40, 60, 80, 100], with the exception of VinDr-CXR where 20% was sampled due to fewer adults having valid age annotations. The calibration set is curated from 10% of the chest x-rays sampled from patients with cardiomegaly, patients without any findings, and patients with a finding other than cardiomegaly. After which, data from patients with a finding other than cardiomegaly are removed. Of the remaining data, 75% is used for training, while the other 25% is used for validation **(Supplementary Table 1)**. All images are loaded in RGB channels and resized to (224, 224). For each of the four adult datasets, we computed dataset-specific image normalization parameters (mean, standard deviation) beforehand. These parameters are then used by the respective models to standardize all images for both training and inference.

	**All**		**Train**		**Validation**		**Calibration**		**Healthy Adults**	
	N	% Positive	N	% Positive	N	% Positive	N	% Positive	N	% Positive
**VinDr-PCXR**	5816	0								
**VinDr-CXR**	14,778	14.39%	9795	14.65%	3265	14.64%	1452	14.67%	266	0
**PadChest**	29,171	18%	18,077	19.61%	6026	19.62%	2678	19.60%	2390	0
**NIH**	20,754	7.20%	12,710	7.94%	4237	7.93%	1884	7.96%	1923	0
**CheXBERT**	6417	36.37%	4064	38.93%	1355	38.89%	593	37.94%	405	0

Supplementary Table 1. Summary statistics of the processed chest x-ray datasets. The percentage of positive Cardiomegaly cases are kept consistent across training, validation and calibration sets.

Perturbation Analysis. To minimize harmful exposure when acquiring chest radiographs, children typically receive lower radiation dosages compared to adults, resulting in images with possibly lower contrast or quality[82,83]. Neural networks may latch onto this low-level difference in pediatric images. In addition to our evaluation, we explored how accounting for these differences between datasets can impact the bias on pediatric patients. Histogram matching is a technique that allows us to adjust the pixel distribution of one image to match the pixel distribution of one or more images. We utilized histogram matching to adjust the pixel distributions of imaged healthy children (aged 0 to 1 years old) in the VinDr-PCXR dataset to match the pixel distributions of images in each of the adult-centric datasets, separately. We varied the strength of the histogram matching across different blending ratios.

medRxiv preprint doi: https://doi.org/10.1101/2025.06.06.25328913; this version posted June 10, 2025. The copyright holder for this preprint (which was not certified by peer review) is the author/funder, who has granted medRxiv a license to display the preprint in perpetuity.
It is made available under a CC-BY 4.0 International license .

Additionally, we plotted pixel histograms for each dataset using random samples of 1000 images (**Supplementary Figure 1**).

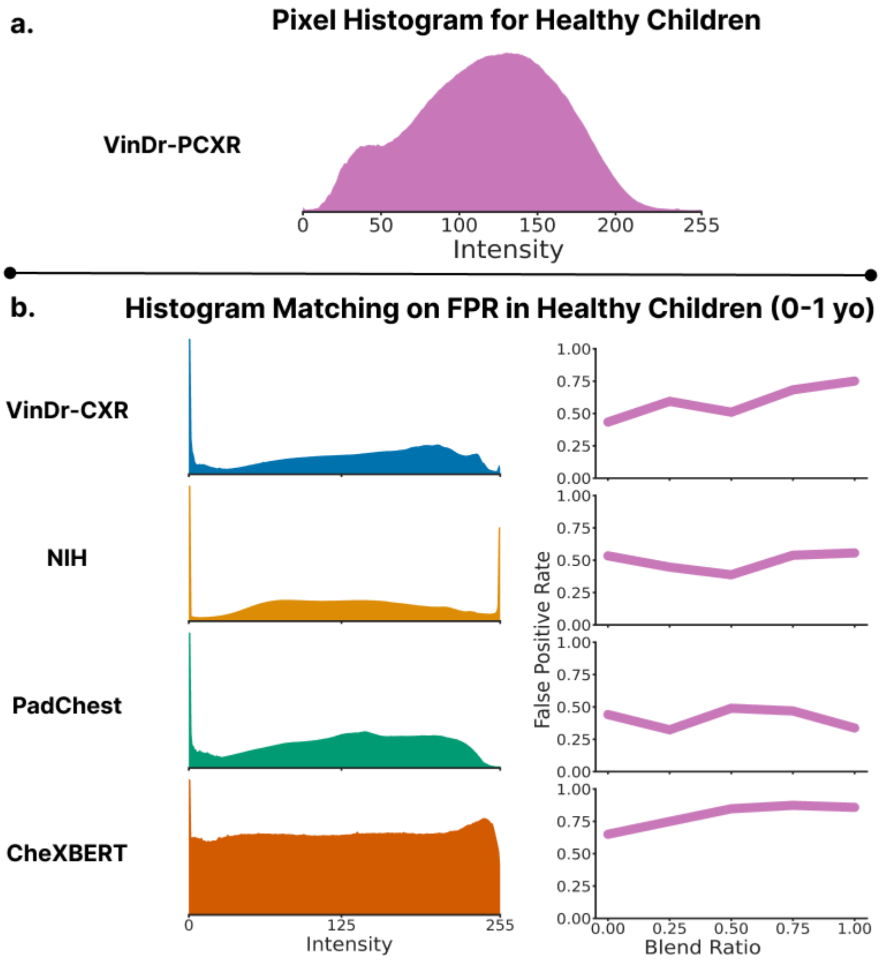

Supplementary Figure 1. **Histogram matching fails to rescue bias against healthy children. (a)** VinDr-PCXR pixel histogram. **(b)** Pixel histograms for adult datasets **(left)** and impact of histogram matching on the model trained on the corresponding adult dataset **(right).**
